# Health-related quality of life and survival in Chinese patients with chronic liver disease

**DOI:** 10.1186/1477-7525-11-131

**Published:** 2013-08-01

**Authors:** Feng Gao, Ru Gao, Guang Li, Zhan Min Shang, Jian Yu Hao

**Affiliations:** 1 Digestive Department, Beijing Chao-Yang Hospital, Capital Medical University, No.8 Gong Ren Ti Yu Chang Nan Road, Chao Yang District, Beijing 100020, China

**Keywords:** Health-related quality of life, Chronic liver disease, SF-36, Survival

## Abstract

**Background:**

To investigate the relationship between health-related quality of life (HRQOL) and survival in Chinese patients with chronic liver disease (CLD).

**Methods:**

HRQOL was measured with the Chinese version of Short Form 36 (SF-36). SF-36 scores, demographic and clinical data were collected at baseline and after 18 months follow-up. Kaplan-Meier and Cox Proportional Hazard Regression survival analyses were used for interpretation of data. Surviving patients were censored in the analyses.

**Results:**

A total of 415 Chinese patients with CLD and 86 healthy controls were enrolled. During the follow-up period 50 patients died. SF-36 scores in healthy controls and surviving patients were higher compared with those in deceased patients. Scores of physical component summary (PCS) in healthy controls, surviving and deceased patients were 54.1 ± 5.2, 48.9 ± 7.7 and 33.5 ± 8.2 respectively (p < 0.001). Scores of mental component summary (MCS) in healthy controls, surviving and deceased patients were 56.6 ± 8.2, 53.0 ± 5.6 and 37.1 ± 12.1 (p < 0.001) respectively. Survival was significantly associated with PCS and MCS scores, and the presence of ascites.

**Conclusions:**

HRQOL was associated with survival in patients with CLD. PCS and MCS scores were predictors of survival.

## Introduction

Chronic liver disease (CLD) is known to cause significant morbidity and mortality, typically due to a number of complications that include ascites, hepatic encephalopathy, variceal hemorrhage and hepatorenal syndrome [1,2]. CLD negatively impacts health-related quality of life (HRQL) [3-6] with patients suffering from fatigue, loss of self-esteem, an inability to function at work, anxiety, depression and other emotional problems that profoundly decrease their quality of life and well-being [7-13]. Although recent research shows that HRQOL scores are independent prognostic factors for overall survival in patients with unresectable hepatocellular carcinoma (HCC) and can predict survival in liver transplant candidates [14-17], the relationship between HRQOL and survival in patients with CLD remains unclear. Therefore, the aim of this study was to examine whether scores from the Short Form 36 (SF-36) health survey are able to predict survival in Chinese patients with CLD.

## Methods

### Ethics

The study received ethics approval from the Ethics Board of Beijing Chao-Yang Hospital, Capital Medical University and all participants gave written informed consent.

### Patient selection

Between September 2009 and September 2011, eligible patients with CLD, aged 18 to 80 years from the Digestive Department of Beijing Chao-Yang Hospital, Capital Medical University were approached to participate in the study. Patients with other chronic active medical diseases (such as congestive heart failure, chronic obstructive pulmonary disease), psychiatric conditions, malignancy, liver transplantation and those unable to communicate or who declined to participate were excluded. Patients with hepatitis B virus (HBV) infection and chronic hepatitis C virus (HCV) infection or who received interferon therapy in the previous 3 months were also excluded. The healthy controls consisted of individuals without chronic disease, aged 18 to 80 years, male or female and who regularly undertook health screening in our hospital’s health examination center. Healthy controls were used to exclude the influence of unpredictable events, social and environmental factors.

Each patient had an established diagnosis confirmed by a Hepatologist. The diagnosis of chronic HBV infection was based on the presence of hepatitis B surface antigen for more than six months, elevated serum alanine aminotransferase levels, with or without HBV DNA as detected by the hybridization method [18]. The diagnosis of chronic HCV infection was based on a positive hepatitis C antibody (ELISA II analysis), elevated serum alanine aminotransferase levels, with or without HCV RNA as detected by polymerase chain reaction [19]. The diagnosis of primary biliary cirrhosis was based on a positive antimitochondrial antibody and elevated liver enzymes with or without liver biopsy [20]. Alcohol was deemed the etiology of chronic liver disease if daily alcohol consumption was greater than 40 g for at least 10 years with elevated γ-glutamyl transferase and exclusion of other liver diseases [21]. The diagnosis of autoimmune hepatitis was based on simplified diagnostic criterion [22]. The diagnosis of liver cirrhosis was based on clinical, biochemical, serologic, ultrasonographic and radiographic parameters.

### Data collection

On admission, patients gave written informed consent and completed the self-administered HRQOL questionnaire. The Medical Outcomes Study of Short Form36 (SF-36 v2 Chinese version) is a widely used and validated generic HRQOL questionnaire. Extensive demographic and clinical data were collected on admission and marital status was dichotomized into single and paired; single was extended to include unmarried persons, divorced or deceased couples.

Laboratory data included alanine and aspartate aminotransferases, alkaline phosphatase, γ-glutamyl transferase, total bilirubin, serum albumin, serum creatine, prothrombin time, serum potassium and sodium, hemoglobin, white blood cell and platelet counts (DADE Dimension Rx1 full-automatic biochemical analyzer).

### HRQOL survey

The SF-36 v2 Chinese Version (from Quality Metric Incorporated) consists of 36 items divided into eight domains that are aggregated into two summary scores, a mental component summary (MCS) and a physical component summary (PCS). These domains range from reflecting predominantly physical wellbeing, that include physical function (PF), the ability to perform expected physical roles (RP), the degree of bodily pain (BP) and overall sense of general health (GH) to those reflecting predominantly social and emotional well-being that include overall sense of vitality (VT), ability to function in social roles (SF), ability to perform expected emotional and social roles (RE) and overall sense of mental health (MH) [23-25].

### Mortality assessment

We followed all participants longitudinally and terminated the follow-up at the time of the patient’s death or March 1st, 2013, whichever occurred first.

### Comparison groups

After the follow-up period, patients were divided into survival and deceased groups.

### Statistical methods

Categorical data were described as the number and continuous data as mean ± SE. Data were analyzed using independent sampled *t* test, one-way analysis of variance (ANOVA) or Chi-square test. Univariate Cox Proportional Hazard Regression analysis was conducted for PCS, MCS, demographic and clinical variables. Variables with a p value <0.1 in univariate Cox Proportional Hazard Regression analysis were entered into multivariate Cox Proportional Hazard Regression analysis. Survival curves were estimated with the Kaplan-Meier method across the quartiles of PCS and MCS scores. A p value <0.05 was considered as statistically significant. All data were analyzed with SAS 9.1.

## Results

### Demographic and clinical data of respondents

A total of 415 Chinese patients with CLD and 86 healthy controls were enrolled and 18 months of follow-up was completed. A total of 50 patients died and two patients were treated with liver transplantation, one in our hospital and one in a different hospital. Both patients died within one month and were therefore excluded from the analysis. The duration of follow-up for surviving and deceased patients was 30.7 ± 10.0 months and 12.1 ± 5.8 months respectively. A further 22 patients were lost to follow up and were also excluded from the analysis. There were no significant difference between the groups for health control and CLD, on age, gender, marital status or educational level (Table 1).

**Table 1:** Demographic data of different groups

**Items**	**Healthy control**	**Chronic liver disease**		**Independent sample *t *test or Chi-square**
	***n*** **= 86 *n *%**	***n*** **= 415 *n *%**		
Age (mean ± SE, yr)	53.1 ± 13.1	54.2 ± 13.4		*P* = 0.441
Male/Female (*n*)	51/35 59/41	243/172 58/42		*P* = 0.898
Paired/Single (*n*)	84/2 98/2	396/19 95/5		*P* = 0.343
Educational level (*n*)				*P* = 0.184
Primary school	10 11	47 11		
Middle school	54 63	273 66		
College or University	18 21	90 22		
College above	4 5	5 1		
Etiologies of chronic liver disease	–	No Cirrhosis *n* %	Cirrhosis *n* %	–
AIH (*n*)	–	16 4	18 5	–
ALD (*n*)	–	62 15	54 13	–
CHB (*n*)	–	54 13	64 15	
CHC (*n*)	–	28 7	36 9	–
PBC (*n*)	–	34 8	35 8	–
Unknown (*n*)	–	–	14 3	–
Present ascites	–	–	158 38	–
Present varices	–	–	196 47	–
Present bleeding	–	–	84 20	–

*AIH* Autoimmune Hepatitis, *ALD* Alcoholic Liver Disease, *CHB* Chronic Hepatitis B, *CHC* Chronic Hepatitis C, *PBC* primary biliary cirrhosis.

### Quality of life and clinical data of respondents

All scores for SF-36 were significantly lower in deceased patients compared with surviving patients with CLD and healthy controls (Table 2). Laboratory results from deceased patients showed significantly lower levels of albumin, hemoglobin, platelet count, serum potassium and serum sodium and significantly higher levels of total bilirubin, prothrombin time, blood urea nitrogen and serum creatinine.

**Table 2:** HRQOL and clinical data of different groups (mean ± SE)

**Items**	**Healthy control**	**Chronic liver disease**		**ANOVA**
		**Surviving patients**	**Deceased patients**	
	***n*** **= 86**	***n*** **= 365**	***n*** **= 50**	
Physical function	93.2 ± 6.4	86.6 ± 15.4	70.1 ± 29.9	*P* < 0.001
Physical roles	88.8 ± 15.6	80.5 ± 19.3	63.0 ± 32.6	*P* < 0.001
Bodily pain	85.1 ± 15.1	87.3 ± 14.2	72.9 ± 26.2	*P* < 0.001
General health	75.3 ± 18.1	56.3 ± 21.3	44.9 ± 28.7	*P* < 0.001
Vitality	83.5 ± 12.0	76.1 ± 10.7	42.7 ± 21.9	*P* < 0.001
Social roles	94.1 ± 10.6	81.0 ± 20.0	39.5 ± 21.0	*P* < 0.001
Emotional roles	92.9 ± 10.7	89.6 ± 13.4	54.4 ± 26.3	*P* < 0.001
Mental health	87.9 ± 25.0	79.3 ± 11.0	52.2 ± 23.4	*P* < 0.001
PCS	54.1 ± 5.2	48.9 ± 7.7	33.5 ± 8.2	*P* < 0.001
MCS	56.6 ± 8.2	53.0 ± 5.6	37.1 ± 12.1	*P* < 0.001
Laboratory data				
Albumin (g/L)	37.0 ± 2.7	35.3 ± 7.4	24.5 ± 4.8	*P* < 0.001
ALT (U/L)	23.0 ± 10.4	56.3 ± 62.8	37.8 ± 31.7	*P* = 0.001
AST (U/L)	23.4 ± 12.7	63.3 ± 69.5	82.7 ± 69.4	*P* < 0.001
GGT (U/L)	51.8 ± 58.0	102.8 ± 146.3	123.9 ± 216.9	*P* = 0.088
ALP (U/L)	105.6 ± 72.0	126.8 ± 94.9	140.7 ± 128.0	*P* = 0.267
TBIL (umol/L)	9.4 ± 3.9	26.8 ± 35.7	81.4 ± 77.1	*P* < 0.001
BUN (umol/L)	5.0 ± 0.9	5.4 ± 2.8	9.9 ± 7.4	*P* < 0.001
Cr (umol/L)	68.8 ± 14.7	77.2 ± 26.0	109.1 ± 65.5	*P* < 0.001
Prothrombin time (s)	10.5 ± 0.5	13.3 ± 2.8	18.3 ± 5.7	*P* < 0.001
WBC (10^9^/L)	6.0 ± 1.4	4.7 ± 2.0	5.6 ± 4.7	*P* < 0.001
Hemoglobin (g/L)	136.0 ± 19.8	118.7 ± 25.4	90.6 ± 20.7	*P* < 0.001
Platelet (10^9^/L)	210.4 ± 35.0	131.1 ± 75.3	77.3 ± 44.5	*P* < 0.001
K (mmol/L)	4.0 ± 0.3	4.0 ± 0.4	3.8 ± 0.7	*P* = 0.041
Na (mmol/L)	140.2 ± 3.5	140.8 ± 7.9	135.7 ± 6.9	*P* < 0.001

*PCS* physical component summary, *MCS* mental component summary, *ALT* alanine aminotransferase, *AST* aspartate aminotransferase, *ALP* alkaline phosphatase, *GGT* γ-glutamyl transferase, *TBIL* total bilirubin, *Cr* serum creatine, *BUN* blood urea nitrogen, *K* serum potassium, *Na* serum sodium, *WBC* white blood cell.

### Cox proportional hazard regression

All variables related to survival with a p value of <0.1 in the univariate analyses were subjected to multivariate analyses. PCS scores, MCS scores and the presence of ascites were significantly associated with survival (Table 3). For a one point increase of PCS, the risk of death was reduced by 0.099. For a point increase of MCS, the risk of death reduced by 0.124. The presence of ascites increased the risk of death by 7.432. Model Fit Statistics (AIC) for multivariate was 369.08 (p < 0.001).

**Table 3:** Results of Cox regression analyses

**Variables**	**Univariate**		**Multivariate**	
	**HR (95% CI)**	***P value***	**HR (95% CI)**	***P value***
Physical function	0.971 (0.961–0.981)	*P* <0.001	–	–
Physical roles	0.973 (0.963–0.983)	*P* <0.001	–	–
Bodily pain	0.964 (0.952–0.977)	*P* <0.001	–	–
General health	0.979 (0.967–0.991)	*P* <0.001	–	–
Vitality	0.911 (0.898–0.925)	*P* =0.001	–	–
Social roles	0.934 (0.921–0.947)	*P* <0.001	–	–
Emotional roles	0.935 (0.924–0.945)	*P* <0.001	–	–
Mental health	0.925 (0.913–0.937)	*P* <0.001	–	–
PCS	0.852 (0.826–0.880)	*P* <0.001	0.901 (0.867–0.935)	*P* <0.001
MCS	0.856 (0.834–0.878)	*P* <0.001	0.876 (0.848–0.905)	*P* <0.001
Age	1.059 (1.034–1.084)	*P* <0.001	–	–
Etiology	1.118 (0.967–1.294)	*P* =0.131	–	–
MELD	1.121 (1.074–1.170)	*P* <0.001	–	–
Ascites	89.883 (12.40–651.0)	*P* <0.001	8.432(1.117–63.63)	*P =* 0.038
ALT	0.992 (0.983–1.000)	*P* = 0.056	–	–
AST	1.003 (1.000–1.006)	*P* = 0.073	–	–
PT	1.197 (1.152–1.243)	*P* <0.001	–	–
TBIL	1.011 (1.008–1.014)	*P* <0.001	–	–
WBC	1.132 (1.045–1.227)	*P* =0.002	–	–
HGB	0.964 (0.954–0.974)	*P* <0.001	–	–
PLT	0.985 (0.979–0.992)	*P* <0.001	–	–
Albumin	0.803 (0.766–0.843)	*P* <0.001	–	–
Cr	1.012 (1.008–1.015)	*P* <0.001	–	–
BUN	1.132 (1.096–1.169)	*P* <0.001	–	–
K	0.451 (0.243–0.837)	*P* =0.011	–	–
Na	0.979 (0.968–0.990)	*P* <0.001	–	–

*PCS* physical component summary, *MCS* mental component summary, *MELD* model for end stage liver disease, *ALT* alanine aminotransferase, *AST* aspartate aminotransferase, *PT* Prothrombin Time, *TBIL* total bilirubin, *Cr* serum creatine, *BUN* blood urea nitrogen, *K* serum potassium, *Na* serum sodium, *WBC* white blood cell. Etiology includes autoimmune Hepatitis, alcoholic Liver Disease, chronic Hepatitis B, chronic Hepatitis C, and primary biliary cirrhosis. Model Fit Statistics (AIC) score of multivariate is 369.08 and *P* <0.001.

### Kaplan-Meier analysis

**Figure 1:**
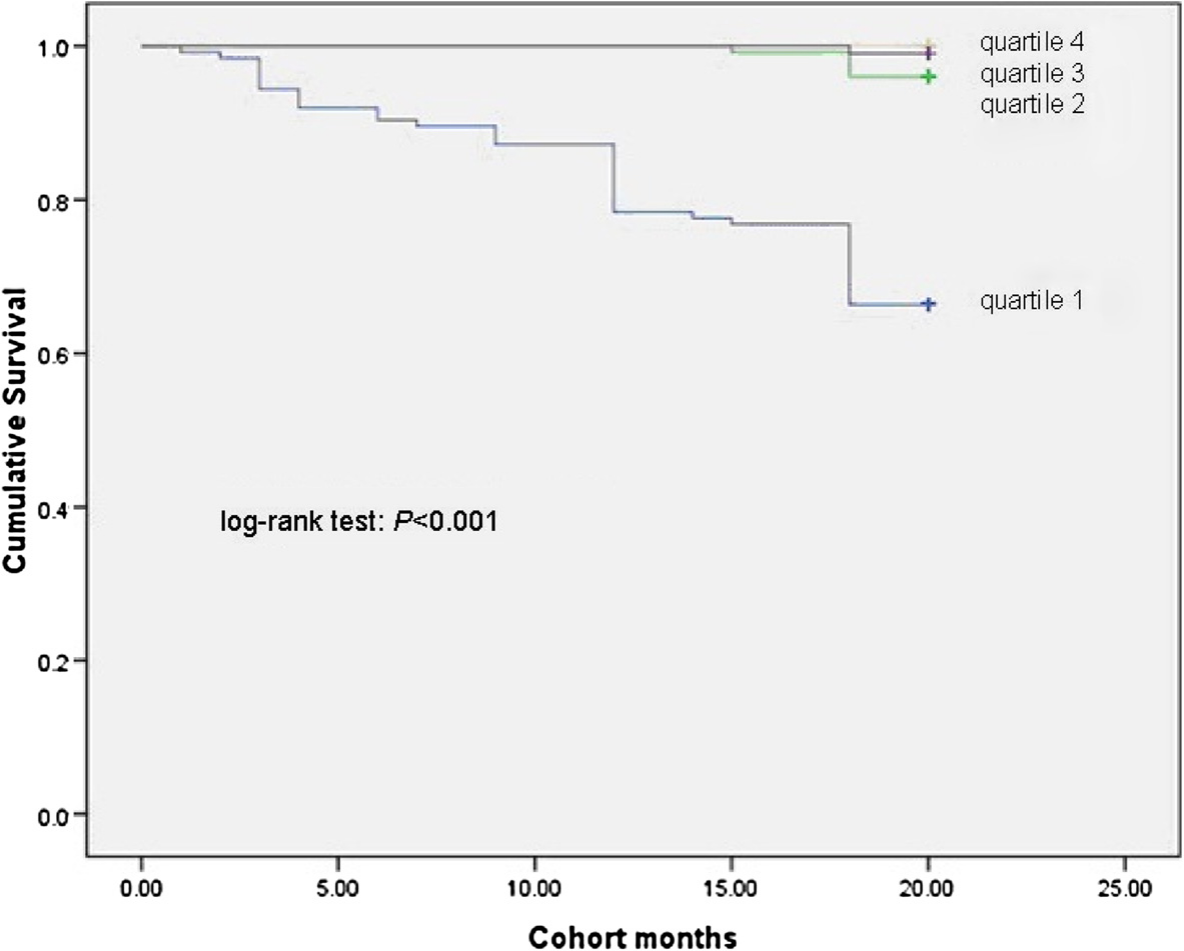
**Kaplan-Meier analysis showing association between PCS scores and survival, log-rank *P*** **< 0.001.**

**Figure 2:**
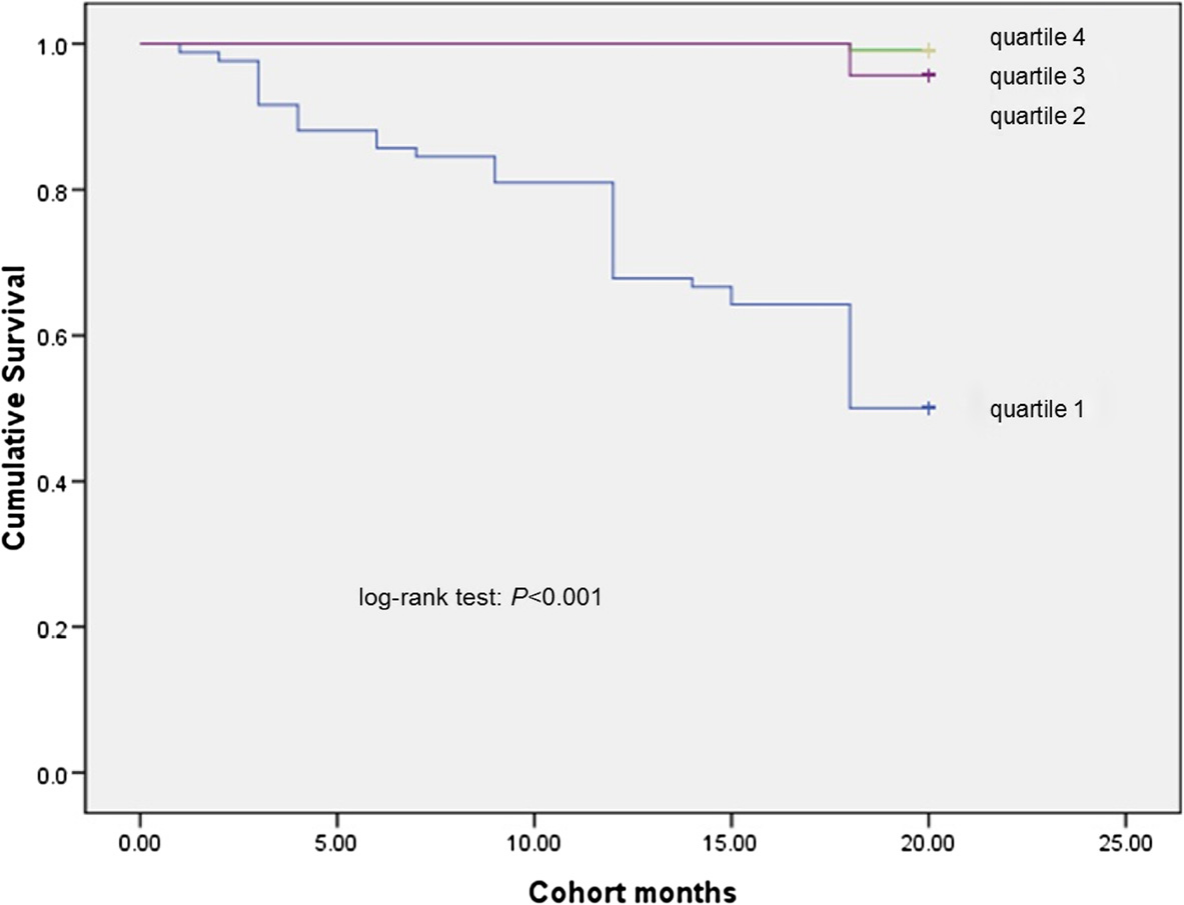
Kaplan-Meier analysis showing association between MCS scores and survival, log-rank p < 0.001.

**Figure 3:**
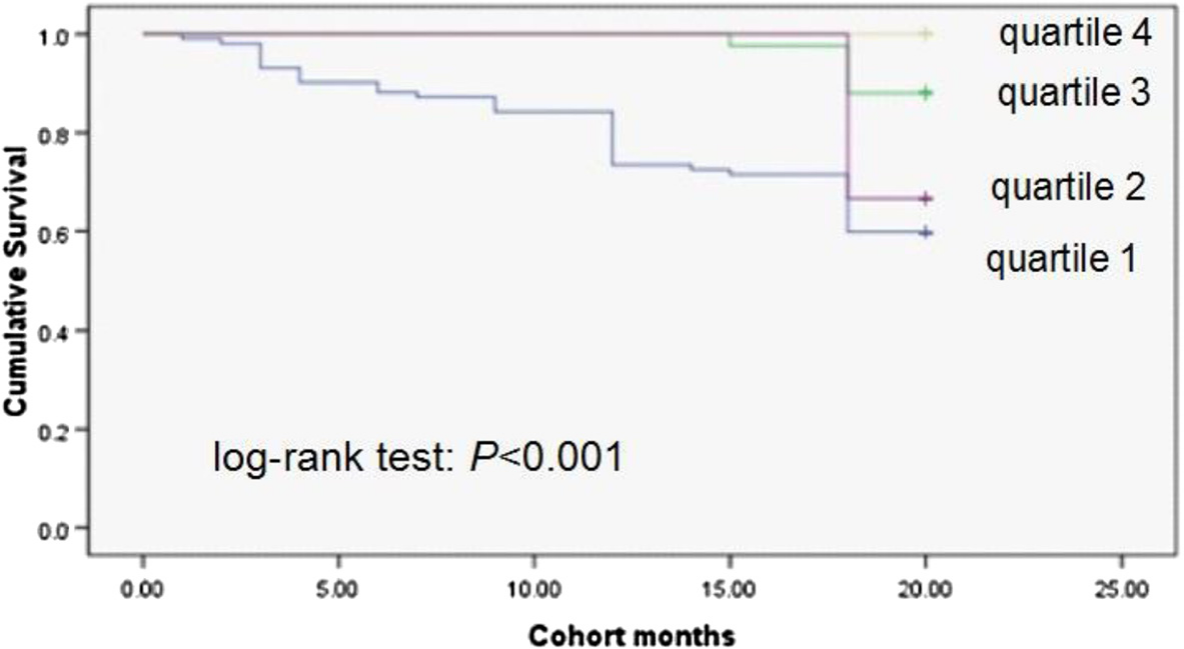
Kaplan-Meier analysis showing association between PCS scores plus present ascites and survival, log-rank p < 0.001.

**Figure 4:**
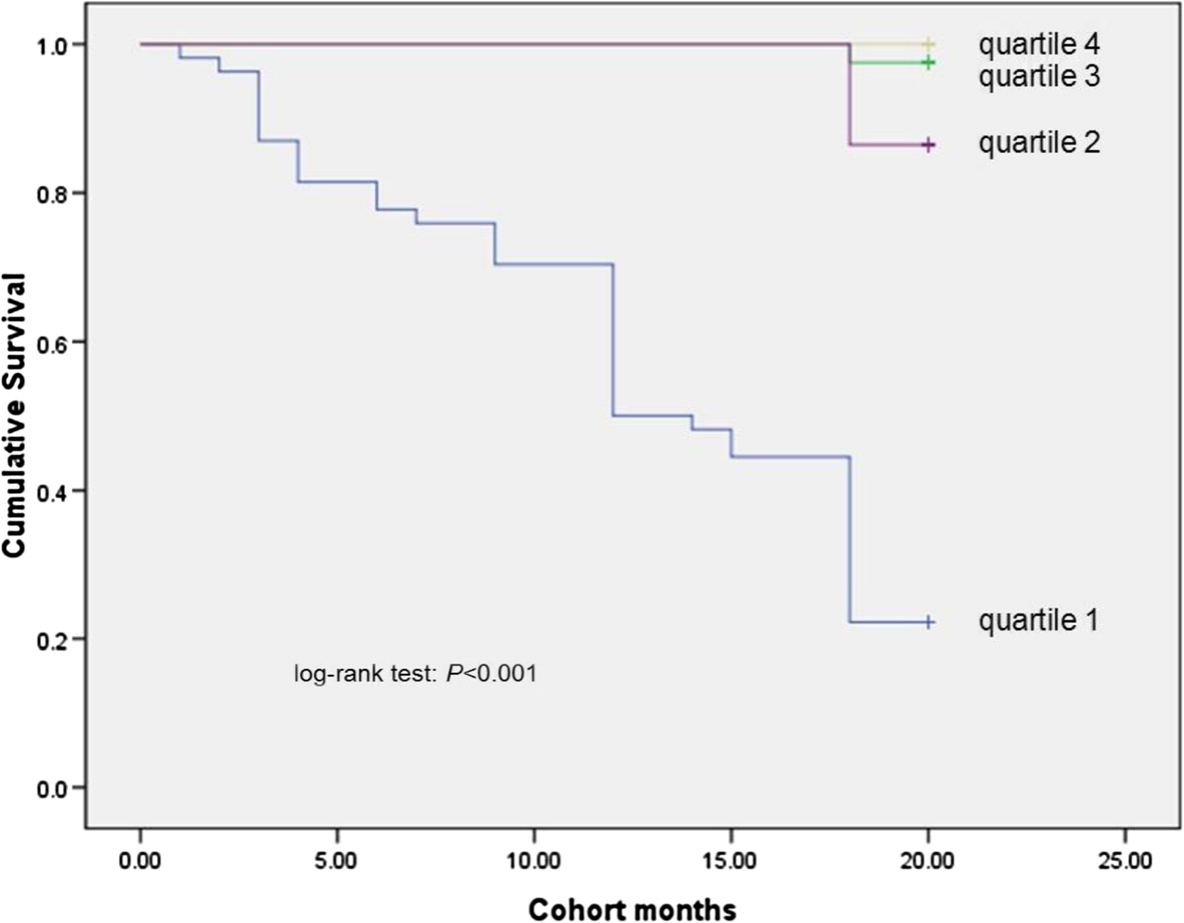
Kaplan-Meier analysis showing association between MCS scores plus present ascites and survival, log-rank p < 0.001.

Survival was significantly different across the quartiles of PCS scores, MCS scores and with the presence of ascites (Figures 1, 2, 3 and 4). Three points of quartile for PCS were 43.5, 50.6, 55.2, and for MCS were 48.9, 53.5, 57.4. Three points of quartile for PCS with the presence of ascites were 33.0, 40.8, 45.8, and for that of MCS were 43.3, 51.2, 55.8.

## Discussion

The results from this study show that deceased patients with CLD had a poor baseline quality of life and a poor clinical state compared with patients who survived. Baseline HRQOL scores were also much worse compared with healthy controls. Recent studies have shown that patients with CLD have substantially reduced HRQOL scores, however, the scores do not differ markedly according to the etiology of the disease. Increasing disease severity is also associated with a poor HRQOL, particularly for the physical component [3-5,10-13]. With progression of liver dysfunction, patients with CLD suffer from fatigue, loss of self-esteem, an inability to function at work, anxiety, depression and other emotional problems that profoundly decrease their quality of life and well-being [23,26]. Furthermore, patients with CLD suffer from complications that reduce their HRQOL, especially for the physical domain area and are less able to maintain daily work and life [23-30]. Our previous study also found that patients with CLD had impaired HRQOL and increasing severity of CLD was associated with a decreasing HRQOL score [31]. Old age, female gender, advanced stage of CLD, presence of ascites, hyperbilirubinemia and a prolonged prothrombin time were important factors in reducing HRQOL.

In this study, patients with higher PCS and MCS scores had a markedly increased survival rate. In a recent report, PCS but not MCS scores from the SF-36 predicted survival [16]. Another report showed that disease-specific HRQOL components, rather than PCS and MCS can be used to identify patients with advanced liver disease who are at high risk of short-term death [17]. There are several possible reasons for this discrepancy. Firstly, the intervention of liver transplantation can markedly improve HRQOL, especially the physical domains, however, in our study, patients only received medical treatment where liver transplantation would have been appropriate, but is limited by funding issues (15 cases per annum receive liver transplantation in our hospital). With progression of liver dysfunction, patients with cirrhosis suffer from fatigue, loss of self-esteem, inability to function at work, anxiety, depression and other emotional problems that profoundly decrease their quality of life and well-being [23,26]. Our previous study showed reduced MCS scores with increasing severity of CLD [31]. Secondly, most patients in our study were relatively stable with a lower mean score of MELD (mean = 7.7) and only 12% died during the follow-up period of 18 months. Therefore, both PCS and MCS were significantly associated with survival.

We found that the presence of ascites was significantly associated with reduced survival, a finding not documented in previous studies even though ascites is the most common complication of advanced CLD. The presence of ascites is regarded as a serious manifestation of advanced CLD and reflects the onset of liver decompensation with an increased mortality (15% in the first year and 44% in the 5 years after diagnosis). Ascites usually becomes refractory to diuretic control with progression of CLD, where survival rates approach 50% at one year [32].

HRQOL measures, such as the SF-36, a generic questionnaire, is used to measure HRQOL in the general population and in patients with chronic diseases including CLD. Many research centers have used HRQOL to compare CLD patients with healthy controls and have generally reported impairment of HRQOL. Additionally, measurement of HRQOL can facilitate integration of biomedical and psychosocial models of health. This integrated approach in the study of patients with CLD will capture the impact of these diseases on patient’s health and well-being [23-30]. Several recent studies have established that better HRQOL is an independent predictor of survival in many chronic diseases such as end-stage renal disease, congestive heart failure, type 2 diabetes and various cancers [33-36]. Further studies also suggest that HRQOL could be used to predict survival of patients with advanced CLD [14,16,17]. Therefore, measurement of HRQOL might compliment objective measures of disease severity to not only accurately and comprehensively assess health status, but also to better stratify risk in patients with CLD.

Our study has a number of limitations. All subjects were recruited from one hospital in one city. Our hospital is a public general affiliated hospital of Capital Medical University and three levels of first-class or China’s most senior hospital in Beijing. Our department has 42 beds including an endoscopy suite. We serve over 150,000 outpatients per year, over 1,800 in-patients per year and over 20000 endoscopies per year that might introduce selection bias. Our sample consisted mostly of patients with health insurance who have better access to healthcare. Therefore, our patient sample excludes patients without health insurance with limited access to care. Our study included patients with CLD with different etiologies and severity. We are addressing these issues in ongoing studies by assessing a larger group of patients in multiple centers.

## Summary

HRQOL was associated with survival in patients with CLD. PCS and MCS scores were predictors of survival.

## Competing interests

The authors declare that they have no competing interests.

## Authors’ contributions

FG participated in the study design, access to data, the data analysis and interpretation, and drafted the manuscript. RG participated in the study design, the data analysis, and important content changes. GL participated in the study design and access to data. ZMS participated in the study design and the data analysis. JYH participated in the study design, the data analysis and interpretation, and important content changes. All authors read and approved the final manuscript.
